# Bacteriological quality of drinking water from source and point of use and associated factors among households in Eastern Ethiopia

**DOI:** 10.1371/journal.pone.0258806

**Published:** 2021-10-15

**Authors:** Yohanis Alemeshet Asefa, Bezatu Mengistie Alemu, Negga Baraki, Dinku Mekbib, Dechasa Adare Mengistu

**Affiliations:** Department of Environmental Health Science, Haramaya University, Harar, Ethiopia; Nanjing University, CHINA

## Abstract

**Background:**

Biological deterioration of drinking water is the major cause of waterborne disease globally. However, there is a paucity of information on identifying the point where deterioration of the bacteriological quality of drinking water occurs (source or point of use) and associated factors among households in developing countries, especially in Ethiopia.

**Method:**

A community based cross-sectional study design was conducted among 425 households in Eastern Ethiopia. Households with at least one child under-five years of age were included in the study. A total of 448 Water samples (425 from households and 23 from water sources) were collected and analyzed by the membrane filtration method to identify Thermotolerant coliform. Binary logistic regression was performed to assess the association between each independent and dependent variable. Adjusted Odd Ratios along with 95% Confidence intervals were estimated to identify factors associated with the outcome variable.

**Result:**

This study revealed that 21.7%; 95% CI (4.5%, 39.1%) of water sources and 83.3%; 95% CI (79.8%, 87.1%) of households’ drinking water were contaminated by thermotolerant coliform. Drinking water samples from households with poor wealth index [AOR = 9.63; 95%CI (2.92, 31.69)], households with unimproved sanitation facility [AOR = 2.81; 95%CI (1.31, 6.01)], households which shares their house with animal [AOR = 3.73; 95%CI (1.66, 8.37)], households that didn’t practice household water treatment [AOR = 3.42; 95%CI (1.60, 7.31)] and not washing hands before water collection [AOR = 7.04; 95%CI (2.22, 22.30)] were significantly associated with deterioration of bacteriological quality of household drinking water.

**Conclusion:**

This study indicates that the bacteriological quality of drinking water deteriorates from source to point of use. Thus, health education programs on water, sanitation, hygienic practice must be enhanced to improve the quality of drinking water.

## Introduction

It is estimated that globally 1.8 billion people use a drinking water source which is contaminated by excreta. Drinking water is found to be more often contaminated in rural areas (41%) than in urban areas (12%) and contamination is most prevalent in Africa (53%) and South-East Asia (35%) [1]. On the other hand, around 525000 under-five children die every year from diarrheal diseases, and majority of these deaths are related to water, sanitation, and hygiene [2, 3]. Pathogens like *Salmonella*, *Vibrio cholera*, *Shigella*, *E*. *coli* O157: H7, *Hepatovirus A* and *Hepatovirus E* are known to be transmitted through contaminated drinking water and cause severe to fatal disease [4].

Different specific microorganisms are used as a marker of drinking water contamination which helps to determine the biological quality of drinking water. The most common microorganisms used to indicate the presence of fecal contamination are Thermotolerant coliform (TTC) and Escherichia coli (*E*. *coli*). Thermotolerant (Fecal) coliform bacteria include the genus Escherichia, and some species of Klebsiella, Enterobacter, and Citrobacter. This group can be detected easily, and it provides an accurate indicator of human health risk. [5].

Different studies in Ethiopia show that the majority of water sources did not fulfill the World Health Organization’s criteria for drinking water quality standards in which 20%to 87.5% of water sources were contaminated by fecal indicator bacteria [6–9]. On the other hand, 58–81% of households drinking water were bacteriologically contaminated [8, 10].

Findings of different studies revealed that the bacteriological quality of drinking water is associated with place of residence, economic status, educational status, type of water sources, household water treatment, hygienic practice, and water storage and handling practice [8–14].

Although different studies in Ethiopia show the existence of microbial contamination of drinking water, most of these studies concentrated on the identification of fecal indicator bacteria only on the water source and very few studies highlighted the point where the contamination of drinking water had occurred (source or point of use). Therefore the main aim of this study is to fill the gap by identifying the bacteriological quality of drinking water from the source and point of use and associated factors among households in Eastern Ethiopia.

## Methods

### Study setting

The study was conducted in Harari Region, Eastern Ethiopia. The Administrative center of Harari region is Harar, which is located at 525km far from Addis Ababa, the capital city of Ethiopia. It is administratively structured into 19 urban and 17 rural kebeles (smallest administrative unit). There were 63,068 (40,904 urban and 22,164 rural) households with a total population of 245,969.

Households in urban kebeles get their drinking water from a groundwater source (borehole with mechanized pump) through a pipeline whereas households in rural kebeles get their drinking water from covered dug well with a hand pump and public tap.

### Study design and procedure

A community-based cross-sectional study was conducted to assess the bacteriological quality of drinking water from source and point of use among 425 households that had at least one child under-five years of age. A stratified sampling technique was used in this study.

In the first stage, kebeles were stratified into two (urban and rural). Thus from stratified 19 urban kebeles, 6 kebeles were selected randomly which had a total population of 13,237households. On the other hand from stratified 17 rural kebeles, 5 kebeles were selected randomly that had a total population of 6,995 households. Then based on population proportion of strata 283 households were allocated from urban households and 149 households were allocated from rural kebeles. Prior to the data collection, a survey was conducted in the selected kebeles to identify households with at least one under-five children and the final households were selected randomly from the survey’s household list.

### Sample size

The sample size was determined using the single population proportion formula with the following assumptions 95% of CI, d (Margin of error) = 4%, 5% for Non-response was used and p = 78% for the proportion of water samples from household water storage contaminated by indicator bacteria [10]. Finally, the minimum sample size required for the study (432) was obtained.

### Data collection

The data were collected using a structured and pretested questionnaire. Initially, it was developed in English, and then translated into local languages, Amharic and Afan-Oromo; then it was translated back into English and administered to the mother or caregiver by local language. Each survey included questions on socio-demographic characteristics, type of water sources, housing and sanitation facilities, household water storage and handling practice, the hygienic practice of respondents and respondent’s knowledge about the causes of drinking water contamination.

Water samples were collected from each household and their respective water source, one at a time, for bacteriological analysis. Sterile 150 ml polyethylene plastic bottles were used for sample collection.

The drinking water in urban kebeles is distributed by a pipeline mainly from groundwater sources (borehole with mechanized pump) found in Dire Dawa, Haramaya, and Erer. So water samples from all functional water sources and 283 households with at least one child under-five years of age that had used these sources were selected randomly. A water sample was collected from each household’s storage container using their drinking cup.

On the other hand, since the rural kebeles got their drinking water from a covered dug well with a hand pump and public tap, 2 water sources were selected randomly from each selected kebele and 149 households with at least one child under-five years of age that had used these sources were selected randomly. A water sample was also selected from each household’s storage container using their drinking cup.

### Operational definition

#### Improved sanitation

A sanitation facilities that includes a flush/pour-flush toilet or latrine that flushes to a sewer, septic tank or pit, a ventilated improved pit (VIP) latrine, pit latrines with the pit well covered by a slab, or composting toilets and must not be shared.

#### Type of water source

In this study, a piped water supply into the dwelling and piped water to a yard/plot is considered as an improved source whereas water supply from covered dug well with a hand pump and a public tab is considered as other improved sources.

#### Sanitary disposal of child feces

The management of a child’s feces by putting or rinsing stools into sanitation facilities.

#### Household water treatment

Are any of a range of devices or methods employed for the purposes of treating water to remove or inactivate microbial pathogens in the home or at the point of use in other settings.

#### Drinking water contamination

Water that has a fecal coliform bacteria detected in any 100ml of the sample.

#### Hand washing facility

The presence of any device or infrastructure that enables a resident to wash their hands effectively using running water, such as a sink with tap, water tank with tap, bucket with tap, tippy tap, or other similar device and both water and soap or detergent must be available at the hand washing facilities.

### Statistical analysis

Data were checked for completeness and coded and double entered into Epi Data version 3.1 and data analysis was performed using SPSS version 22.0. Descriptive statistics which involved frequency and percentage for the dependent and independent variables were used. Categorical variables were expressed as number (percentage, %). After laboratory analysis contaminated water samples from households (a water sample that contains one or more TTC were coded as ‘1’ and water samples with no TTC were coded as ‘0’). A chi-square test was used to evaluate the differences in the distribution of categorical variables for study groups. The family wealth index was constructed using Principal Component Analysis (PCA) method by considering locally available household assets and the family wealth was categorized into terciles.

A multivariable logistic regression model was fitted to identify the association between independent variables and the bacteriological quality of drinking water. First, bivariate analysis was done to identify candidate variables for multivariable logistic regression. Second, to identify predictors of bacteriological quality of drinking water having a p <0.25 was entered in the multivariable logistic regression model [15, 16]. At this step, the interaction between different independent variables was checked and Collinearity diagnostics was done by checking the standard error less than two. All statistical analysis was set at 5% level of significance (i.e. p < 0.05). Multivariate Analysis with Adjusted Odds Ratio (AOR) was used to control possible confounders and to determine factors associated with the bacteriological quality of drinking water among households.

### Ethical statement

Ethical clearance was obtained from the Institutional Health Research Ethical Review Committee (IHRERC) of Haramaya University College of Health and Medical Sciences. Consent was obtained from district administration, district health department, and water supply and sewerage authority. Individual Informed written consent was obtained from each study participant. The respondents were assured of confidentiality by excluding their names during the data collection. They were informed well that they had the full right to refuse to participate or withdraw from the study at any time without any prerequisite.

### Laboratory analysis

All water samples were transported to Haramaya University laboratory using ice packs and reached to the laboratory within 4 hours of collection. The membrane filtration method was used to identify the indicator bacteria (Thermotolerant coliform bacteria). The samples were subsequently passed through a 0.45-micrometer pore and the filter paper with its medium was placed in an incubator at 44°c for 18 hours to be analyzed [17]. One blank sample (using boiled dilution water) was analyzed for every 10 water samples to make sure that there is no secondary contamination [18].

## Results

### Socio-demographic and economic characteristics

In this study, a total of 425 households were participated making a response rate of 98.3%. From this 279 (65.6%) were living in urban. The mean ages of the respondents were 30 years. Majority 276 (64.9%) of participants were Muslim religion followers. Two hundred forty (56.5%) of the participants were housewives by occupation and 157(36.9%) were unable to read and write. Besides, 170 (40%) of the households had a family size of more than 5 (S1 Table).

### Type of water source and accessibility of water

Piped water in the yard was the main source of drinking water among the majority of households 229 (53.9%) followed by protected well 126(29.6%) and public tap 58(13.6%). Majority of urban (82.1%) and rural (86.3%) households got their water from piped water in the yard and protected well respectively. The proportion of households that meet the criteria for basic water accessibility in-terms of time (less than 30 minutes round trip) and quantity (>20 liters /capita per day) were 353 (83.1%) and 66 (15.5%) respectively. From these, 96.4% of urban households got their drinking water within 30 minutes roundtrip compared to only 57.5% of rural households. Majority 359 (84.5%) of the households also pay for water.

### Household water storage and handling practices

Concerning water storage, 378 (88.9%) of the households was used plastic container (Jerrycan) to collect and store their drinking water and also used a pouring method to withdraw water from their container. During the time of data collection 77(18.1%) of water storage containers did not have cover. Out of the total 425 households, nearly half 208 (48.9%) of the households’ storage container were accessible to children and 152(35.8%) of them place their drinking cups on the floor. with regard to water treatment only 70 (16.5%) of them used household water treatment and from this majority 49 (70%) of them use chlorine to treat their water. From this, only 56 (20%) of urban households and 14 (9.6%) of rural households practiced household water treatment. Regarding the knowledge of respondents about the cause of water contamination 337 (79.3%) of the respondents had good knowledge and the majority of the respondents 354 (83.3%) did not receive any Water sanitation and hygiene (WaSH) education in the past 3 months (S2 Table).

### Housing and sanitation facility

Regarding sanitation facility, 363(85.4%) of the households had latrine with the majority 216 (50.8%) of them had used simple pit latrine without cover. From those households that had latrine 181(49.9%) of them were sharing their latrine with other households. Concerning the child feces disposal 341 (80.2%) of the respondents practice sanitary disposal. About 336 (79.1%) of the participants also disposed their solid waste by communal collection, burning, dumping in the waste pit or by composting. In addition, about 157 (36.9%) of households shared their house with domestic animals.

### Hygienic practice

From 425 responding households about 370 (87.1%) of them wash their water storage container regularly and 320 (75.3%) of them also wash their hand before water collection. In addition to this, 291 (68.5%), 304 (71.5%) and 297 (69.9%) of the respondents washed their hands with soap after visiting the toilet, after cleaning child and before feeding a child respectively. Beside this 38 (8.9%) of the households had a place for hand washing, but only 30 (7.1%) of them had water and cleaning agent like soap during the time of data collection (observation) (S3 Table).

### Bacteriological quality of drinking water sources

A water sample from different points were taken once and analyzed by membrane filtration method. The sources included in the study were 13 boreholes with mechanized pump (deep wells), 8 covered dug wells with hand pump (shallow well) and 2 public tap. The result indicates that 5 (21.7%); 95% CI (4.5%, 39.1%) of the total sources were contaminated by Thermotolerant coliform (TTC) and the mean thermotolerant coliform (TTC) were 1.04colonies/100ml (Table 1).

**Table 1:** Bacteriological quality of drinking water sources, Eastern Ethiopia.

Type of source	N	Bacteriological quality		
		Contaminated	Not contaminated	Mean number of TTC colonies/100ml
		Freq (%)	Freq (%)	
Borehole with mechanized pump (Deep wells)	13	-	13(100%)	-
Covered dug well with a hand pump (Shallow wells)	8	4(50%)	4(50%)	5.25
Public tap	2	1(50%)	1(50%)	3
**Total sample**	**23**	**5(21.7)**	**18(78.3)**	**1.04**

### Bacteriological quality of the households’ drinking water

From households that had used the above sources, water samples were taken for bacteriological analysis from their storage container using their drinking cup. The bacteriological quality analysis of drinking water from the households’ storage container using their drinking cup indicates that 354(83.3%); 95% CI (79.8%, 87.1%) were contaminated by thermotolerant coliform with mean thermotolerant coliforms of 84.42 colonies per 100ml (Table 2).

**Table 2:** Bacteriological quality of households’ drinking water, Eastern Ethiopia.

Bacteriological quality of households’ drinking water	Frequency	Percentage
Contaminated	354	83.3%
Not Contaminated	71	16.7%
**Total Sample**	**425**	**100%**

### Factors associated with bacteriological quality of drinking water among households

Bivariate and multivariate analysis was done in the binary logistic regression to detect factors associated with the bacteriological quality of drinking water. In the multivariate logistic regression analysis, the family wealth index, type of sanitation facility, sharing the house with animals, household water treatment and washing hands before water collection were statistically significant at 5% level of significance and were found to be the predictors of bacteriological quality of drinking water among the households.

The odds of household water contamination were 9.63 times higher among households in poor tercile than rich [AOR = 9.63; 95%CI (2.92, 31.69)]. Households with unimproved sanitation facilities had 2.81 times higher odds of contaminated water relative to those with improved sanitation facilities [AOR = 2.81; 95%CI (1.31, 6.01)]. A drinking water sample from households which shares their house with animal were 3.73 times more likely to be contaminated when compared with households that didn’t share their house with animals [AOR = 3.74; 95%CI (1.66, 8.37)].

Drinking water from households that didn’t practice household water treatment was also 3.42 times more likely to be contaminated than those households that practiced household water treatment [AOR = 3.42; 95%CI (1.60, 7.31)]. Besides, washing hands before water collection was also predicting the bacteriological quality of drinking water. Drinking water samples from respondents who did not wash their hands before water collection were 7 times more likely to be contaminated than those who wash their hands before water collection [AOR = 7.04; 95%CI (2.22, 22.30)] (Table 3).

**Table 3:** Factors associated with bacteriological quality of drinking water among households, Eastern Ethiopia.

Covariates	Category	Bacteriological Contamination of drinking water		COR (95% CI)	AOR (95% CI)
		Yes	No		
Educational status of the respondents	College and above	27	20	0.13(0.06, 0.29)	0.40(0.13, 1.23)
	Formal education	162	28	0.56(0.28, 1.11)	1.60(0.63, 4.04)
	Informal education	22	9	0.23(0.09, 0.61)	0.33(0.10, 1.15)
	Unable to read and write	143	14	1	1
Family wealth index	Poor	125	8	9.12(4.13, 20.16)	9.63(2.92, 31.69)*
	Medium	140	11	7.43(3.68, 15.01)	7.39(3.05, 17.89)*
	Rich	89	52	1	1
Place of residence	Rural	135	11	3.36(1.70, 6.62)	0.11(0.01, 1.42)
	Urban	219	60	1	1
Type of water source	Piped	182	59	0.21(0.11, 0.41)	0.18(0.01, 1.83)
	Other improved sources	172	12	1	1
Type of sanitation facility	Unimproved	312	44	4.55(2.55, 8.12)	2.81(1.31, 6.01)*
	Improved	42	27	1	1
Sharing the house with animals	Yes	146	11	3.82(1.94, 7.53)*	3.73(1.66, 8.37)*
	No	208	60	1	1
Child feces disposal	Sanitary disposal	275	66	0.26(0.10, 0.67)	0.93(0.26, 3.28)
	Unsanitary disposal	79	5	1	1
Household water treatment	Yes	45	25	1	1
	No	309	46	3.73(2.09, 6.65)	3.42(1.60, 7.31)*
Washing hands before water collection	No	101	4	6.68(2.37, 18.82)*	7.04(2.22, 22.30)*
	Yes	253	67	1	1
Availability of hand washing facility	No	331	56	3.85(1.89, 7.83)	1.80(0.68, 4.73)
	Yes	23	15	1	1

CI = Confidence Interval, COR = Crude Odds Ratio, AOR = Adjusted Odds Ratio.

* = p-value < 0.05.

## Discussion

This study revealed that 21.7%; 95% CI (4.5%, 39.1%) of water sources and 83.3%; 95% CI (79.8%, 87.1%) of households drinking water were contaminated by thermotolerant coliform. Family wealth index, type of sanitation facility, sharing the house with animals, household water treatment and washing hands before water collection were identified as predictors of the bacteriological quality of drinking water among households.

The present study showed that 21.7% of water sources were contaminated with thermotolerant coliform. The proportion of contaminated water sources was much lower when compared with the result from Fogera and Mecha district of Northwest Ethiopia (73.7%) [8]. This may be due to the reason that all the sources included in this study were improved.

The proportion of households with contaminated drinking water 83.3% (95% CI: 79.8%-87.1%) in the region was closer to that reported in Kote town, India (80%) [19], Tamale Metropolis, Ghana (83%) [20] and Eastern Ethiopia (81%) [10]. On the contrary, this finding reports a higher proportion of households with contaminated drinking water compared to the results of a study done in Fogera and Mecha district of Northwest Ethiopia (58%) [8] and Jigjiga city (55%) [6]. This discrepancy might be related to the method of laboratory analysis in which these studies identify the specific indicator bacteria *E*. *coli*, which is the subset of thermotolerant coliform and this result shows that the bacteriological quality of drinking water deteriorates from source to household.

The present study affirmed that drinking water from poor households was more likely to be contaminated than rich households. This is in line with a result from Nagpur, India in which samples were taken from households with a lower socioeconomic status contain high thermotolerant coliform compared with higher socioeconomic status [21, 22]. This can be explained by the fact that inhabitants from households with poor terciles are hardly to get an adequate amount of water from a pipe in their premises and this will make the difficult situation to protect their domestic and personal hygiene which in turn increases the risk of drinking water contamination.

Households’ type of sanitation facility was also another factor that predicted their bacteriological quality of drinking water in this study. Drinking water samples from households that had used unimproved sanitation facilities had 2.81 times higher odds of contamination than those households with improved sanitation facilities. Similar studies from Ghana [14] and Bagamoyo, Tanzania [23] also reported that water samples from households with unimproved sanitary facilities had higher odds of contaminated water. This may be due to the reason that unimproved sanitation facilities do not protect the environment from contamination.

In this study, a statistically significant association was found between the covariate sharing the house with animals and the bacteriological quality of drinking water in households. Households that share their house with animals had 3.73 times more likely to had contaminated drinking water when compared with households that didn’t share their house with animals. This had been also supported by the study from Nepal [24] and Fogera and Mecha district of Northwest Ethiopia [8]. This association could be due to the fact that fecal indicator bacteria are present in both human and non-human animal feces.

This study showed that drinking water from households that didn’t practice household water treatment was 3.42 times more likely to be contaminated than those households that practice household water treatment. This was consistent with the study done in Eastern Ethiopia [10]. This could be explained by the fact that treating water at the point of use will help to improve the quality of drinking water by attacking the microorganisms and the risk of contamination could also be prevented afterward if chlorine is used for treatment.

A statistically significant association between washing hands before water collection and bacteriological quality of household drinking water was seen in this study. Drinking water samples from respondents who did not wash their hands before water collection was more likely to be contaminated than those who had washed their hands before water collection. This is in line with the result from Tehuldere woreda, Northeast Ethiopia [25] and Fogera and Mecha district of Northwest Ethiopia [8]. This might be due to the reason that one of the pathways of fecal bacteria is from the contact between fingers and feces during defecation or cleaning the child, so washing hands at critical times and before water collection are the best method to remove pathogens.

Social desirability bias is one of the limitations of this study since a self-reported method of data collection was used for socio-demographic, environmental and behavioral factors. This was minimized by adding an observation besides the interview. A false-positive result due to cross-contamination is also another limitation and this was minimized by sterilizing the kit using 99.9% Methanol for 15 minutes. Besides, laboratory analysis of one blank sample for every 10 water samples was done and all the blank samples were negative. On the other hand, since the data in this study is one time data it doesn’t show the seasonal patterns of the bacteriological quality of drinking water in the study area.

## Conclusion

The result of this study revealed that the bacteriological quality of drinking water deteriorates from source to point of use. High proportions of households’ drinking water samples were contaminated by Thermotolerant coliform. Furthermore, family wealth index, sharing the house with animals, type of sanitation facility, household water treatment and washing hands before water collection were associated factors of bacteriological quality of drinking water among households. Therefore, the regional health office should strengthen health education programs on water, sanitation, hygienic practice and proper storage and handling of drinking water to improve the quality of drinking water at the point of use. Additionally, the water supply and sewerage authority should also monitor the bacteriological quality of drinking water at the source, distribution, and household regularly.

## Supporting information

S1 Table Socio-economic and demographic characteristics of households, Eastern Ethiopia. (DOCX)

S2 Table Household water storage and handling practice, Eastern Ethiopia. (DOCX)

S3 Table Hygienic practice of the respondents, Eastern Ethiopia. (DOCX)

S1 File English version questionnaire. (PDF)

S2 File Amharic version questionnaire. (PDF)

S3 File Afaan Oromoo version questionnaire. (PDF)

S1 Dataset The dataset used for the study. (SAV)
