# Supplementary material for: Bacteriological quality of drinking water from source and point of use and associated factors among households in Eastern Ethiopia
Source: PLoS One. 2021 Oct 15;16(10):e0258806. doi: 10.1371/journal.pone.0258806 (PMC8519474; doi:10.1371/journal.pone.0258806)
Supplement: S1 Table — (DOCX) [file pone.0258806.s001.docx]

**Table 1 Socio-economic and demographic characteristics of households, Eastern Ethiopia.**

| **Variables** | **Category** | **Frequency** | **Percentage (%)** |
| --- | --- | --- | --- |
| Age of respondents | 18-29 | 224 | 52.7 |
|  | 30-39 | 145 | 34.1 |
|  | 40-49 | 30 | 7.1 |
|  | > 50 | 26 | 6.1 |
| Religion of respondent | Muslim | 276 | 64.9 |
|  | Orthodox | 126 | 29.6 |
|  | Protestant | 20 | 4.7 |
|  | Others* | 3 | 0.7 |
| Educational level of respondent | Unable to read and write | 157 | 36.9 |
|  | Able to read and write | 31 | 7.3 |
|  | Primary level | 110 | 25.9 |
|  | Secondary level | 80 | 18.8 |
|  | College and above | 47 | 11.1 |
| Current marital status of the respondent | Married | 301 | 70.8 |
|  | Single | 101 | 23.8 |
|  | Divorced | 15 | 3.5 |
|  | Widowed | 8 | 1.9 |
| Occupation of respondent | Unemployed (housewives) | 240 | 56.5 |
|  | Daily laborer | 68 | 16 |
|  | Merchant | 64 | 15.1 |
|  | Government employee | 45 | 10.6 |
|  | Private employee | 8 | 1.9 |
| Family size | < 5 | 255 | 60 |
|  | > 5 | 170 | 40 |
| Wealth index | Poor | 133 | 31.3 |
|  | Medium | 151 | 35.5 |
|  | Rich | 141 | 33.2 |
| Others* (Jehovah’s witness=2, Catholic=1),  Wealth index categorized based on PCA (principal component analysis) | | | |
