# Supplementary material for: Bacteriological quality of drinking water from source and point of use and associated factors among households in Eastern Ethiopia
Source: PLoS One. 2021 Oct 15;16(10):e0258806. doi: 10.1371/journal.pone.0258806 (PMC8519474; doi:10.1371/journal.pone.0258806)
Supplement: S2 Table — (DOCX) [file pone.0258806.s002.docx]

**Table2: Household water storage and handling practice, Eastern Ethiopia, 2018.**

| **Variables** | **Category** | **Frequency** | **Percentage (%)** |
| --- | --- | --- | --- |
| Type of storage container | Jerrican | 378 | 88.9 |
|  | Bucket | 47 | 11.1 |
| Cover for the storage container | Yes | 348 | 81.9 |
|  | No | 77 | 18.1 |
| Method to withdraw water from the storage container | Pouring | 378 | 88.9 |
|  | Dipping | 47 | 11.1 |
| If dipping does the utensil has a handle? | Yes | 22 | 46.9 |
|  | No | 25 | 53.2 |
| Accessibility of storage container by children | Yes | 208 | 48.9 |
|  | No | 217 | 51.1 |
| Regular placement of drinking cups | Place prepared for it | 212 | 49.9 |
|  | On the floor | 152 | 35.8 |
|  | On the water storage container | 61 | 14.4 |
| Method of treatment | Chlorine (bleach) | 49 | 70 |
|  | Strain it through a cloth | 13 | 18.6 |
|  | Boiling | 6 | 8.6 |
|  | Let it stand and settle | 2 | 2.9 |
