# Supplementary material for: Bacteriological quality of drinking water from source and point of use and associated factors among households in Eastern Ethiopia
Source: PLoS One. 2021 Oct 15;16(10):e0258806. doi: 10.1371/journal.pone.0258806 (PMC8519474; doi:10.1371/journal.pone.0258806)
Supplement: S3 Table — (DOCX) [file pone.0258806.s003.docx]

**Table 3: Hygienic practice of the respondents, Eastern Ethiopia.**

| **Variables** | **Categories** | **Frequency** | **Percentage (%)** |
| --- | --- | --- | --- |
| Wash water storage container regularly | Yes | 370 | 87.1 |
|  | No | 55 | 12.9 |
| How frequent the water storage container had been washed | Daily | 119 | 32.2 |
|  | Every 3 days | 16 | 4.3 |
|  | Every 7 days | 194 | 52.4 |
|  | Every 15 days | 35 | 9.5 |
|  | Every month | 6 | 1.6 |
| Wash hands before water collection | Yes | 320 | 75.3 |
|  | No | 105 | 24.7 |
| Wash hands after visiting a toilet | Wash with soap | 291 | 68.5 |
|  | Wash without soap | 78 | 18.4 |
|  | Did not wash | 56 | 13.2 |
| Wash hands after cleaning a child | Wash with soap | 304 | 71.5 |
|  | Wash without soap | 80 | 18.8 |
|  | Did not wash | 41 | 9.6 |
| Wash hands before feeding a child | Wash with soap | 297 | 69.9 |
|  | Wash without soap | 79 | 18.6 |
|  | Did not wash | 49 | 11.5 |
| A place for washing hands with water and cleaning agent | Yes | 30 | 7.1 |
|  | No | 395 | 92.9 |
