# Supplementary material for: Bacteriological quality of drinking water from source and point of use and associated factors among households in Eastern Ethiopia
Source: PLoS One. 2021 Oct 15;16(10):e0258806. doi: 10.1371/journal.pone.0258806 (PMC8519474; doi:10.1371/journal.pone.0258806)
Supplement: S1 File — (PDF) [file pone.0258806.s004.pdf]

English version questionnaire

Kebele: ________________________ Household ID _______________

Name of Data Collector: _________________ Signature___________ Date: _________

Part I: Socio-Economic and demographic characteristics

S. No	Variable	Response	Skip
101	Head of the household	1. Male<br>2. Female	
102	Age of respondent:	_________	
103	Sex of Respondent	1. Male<br>2. Female	
104	Ethnicity of the respondent	1. Oromo<br>2. Amhara<br>3. Harari<br>4. Gurage<br>5. Other specify ____________	
105	Religion of the respondent	1. Muslim<br>2. Orthodox<br>3. Protestant<br>4. Other specify ____________	
106	Educational level respondent	1. Unable to read and write<br>2. Able to read and write<br>3. Grade 1-8<br>4. Grade 9-12<br>5. College and above	
107	Current Marital status of respondents	1. Single<br>2. Married<br>3. Divorced<br>4. Widowed<br>5. Others (specify) ___________	

108	Occupation of respondents	1. Merchant<br>2. Government employee<br>3. Daily laborer<br>4. Unemployed /not working/pensioned<br>5. Other specify___________	
109	Number of individuals in the household	______________	
110	Place of Residence	1. Rural 2. Urban	

Part II: Types of water source

201	What is the immediate water source that is used by the household	1. Piped water into dwelling<br>2. Piped water to yard<br>3. Public tap<br>4. Protected well<br>5. Protected spring<br>6. Other specify _______________	If 1 or 2, to Q204
202	How long does it take to go there, get water and come back?	___________ in minutes	
203	Who usually goes to this source to fetch the water for your household?	3. Adult woman<br>4. Adult man<br>5. Female child (under 15 years)<br>6. Male child (under 15 years)	
204	On average what is the per capita consumption of water for all purposes per day?	___________ liters	
205	Do you pay for water?	1. Yes<br>2. No	

Part III. Household water storage and handling

301	How frequent do you collect water?	1. Every day<br>2. Every other day<br>3. Every 3 days<br>4. Every week<br>5. Others specify __________	
302	For how long do you store drinking water	______________Days	
303	Type of water storage container	1. Jerican<br>2. Bucket<br>3. Other specify ______	
304	Does it have a cover (Observe)	3. Yes<br>4. No	
305	Do you wash your storage container regularly	1. Yes<br>2. No	If 2, to Q 307
306	How frequently do you wash the storage container	1. Daily ____<br>2. Every 3 days<br>3. Weekly<br>4. Every 15 days<br>5. Never	
307	What method do you use to withdraw water from container	1. Pouring<br>2. Dipping	If 1, to Q 309
308	If Dipping does the utensil used to draw water from the container has a handle	1. Yes<br>2. No	
309	Does the storage containers accessible to children	1. Yes<br>2. No	
310	Where do you regularly place cups used for drinking	1. On the floor<br>2. Place prepared for it<br>3. On the water container<br>4. Other specify ___________	

Part IV: Housing and Sanitation facilities

401	What kind of toilet facility do members of your household usually use	1. Pour flush<br>2. Ventilated improved pit latrine (VIP)<br>3. Pit latrine<br>4. No facilities or bush or field<br>5. Others specify ________	
402	Do you share this facility with other households?	1. Yes<br>2. No	If 2, to Q 404
403	How many other households share this toilet?	____________household	
404	Where do you dispose child feces?	1. Child use latrine<br>2. Put/rinsed in to the latrine<br>3. Left open<br>4. Throw into the garbage<br>5. Other specify __________	
405	Where the households usually dispose the solid waste?	1. Open space<br>2. Dump in waste pit<br>3. Burning<br>4. Composting<br>5. Others specify _________	
406	Where the households usually dispose the liquid waste?	1. Soak pit<br>2. Latrine<br>3. Flash away<br>4. Others specify _________	
407	Do animals share the house?	1. Yes<br>2. No	

Part V- Hygienic Practice

501	Do you wash your hands before water collection?	1. Yes<br>2. No	If 2, to Q 503
502	Do you wash your hand after visiting a toilet?	1. Yes<br>2. No	If 2, to Q 505

503	If yes to Q502 do you use a soap to wash your hands?	1. Yes<br>2. No	
504	Do you wash your hand after cleaning a child	1. Yes<br>2. No	If 2, to Q 507
505	If yes to Q504 do you use a soap to wash your hands?	1. Yes<br>2. No	
506	Do you wash your hands before feeding children?	1. Yes<br>2. Sometimes<br>3. No	If 3, to 508
507	If yes to Q506 do you use a soap to wash your hands?	1. Yes<br>2. No	
508	Is there a place for washing hands? (observe)	3. Yes<br>4. No	If 2, to Q601
509	Is the water available? (observe)	1. Yes<br>2. No	
510	Is there any soap or cleansing agent like ash? (observe)	1. Yes<br>2. No	

Part VI: Household water treatment

601	Does the family/household use any water treatment?	1.Yes<br>2. No	
602	If Yes to above question , which method	1. Add bleach/ chlorine<br>2. Strain it through a cloth<br>3. Let it stand and settle<br>4. Boiling<br>5. Others specify __________	

Part VII: Knowledge on the causes of drinking water contamination

701	Where do you think drinking water contamination occurs?	1. At source<br>2. In household<br>3. Both<br>4. Others specify	
702	Do you think cleansing water collection and storage material prevent water contamination?	1. Yes<br>2. No	
703	Do you think washing hands before water collection can prevent water contamination?	1. Yes<br>2. No	
704	Do you think washing hands after defecation can prevent water contamination?	1. Yes<br>2. No	
705	Do you think proper utilization of latrine can prevent water contamination?	1. Yes<br>2. No	
706	Do you think treating water in household can prevent water contamination?	1. Yes<br>2. No	
707	Do you receive WASH education in the last months	1. Yes<br>2. No	

Part VIII: Questions to assess the current family wealth (Economic condition)

Could you tell me if you have the following in your house?

**Asset type**	**Response**	
**Domestic animals**		
Ox	No (0)	Yes (1)
Cow	No (0)	Yes (1)
Calf	No (0)	Yes (1)
Sheep	No (0)	Yes (1)
Goat	No (0)	Yes (1)
Horse	No (0)	Yes (1)
Donkey	No (0)	Yes (1)
Cock and Hen	No (0)	Yes (1)

**Durable assets**		
Television	No (0)	Yes (1)
Radio	No (0)	Yes (1)
Electricity	No (0)	Yes (1)
Refrigerator	No (0)	Yes (1)
Conventional telephone	No (0)	Yes (1)
Mobile phone	No (0)	Yes (1)
Car	No (0)	Yes (1)
Motorcycle	No (0)	Yes (1)
Cycle	No (0)	Yes (1)
Cart	No (0)	Yes (1)
Gold, money	No (0)	Yes (1)
Ownership of owned living house	No (0)	Yes (1)
Ownership of agricultural land	No (0)	Yes (1)
**Productive assets**		
Plough plow	No (0)	Yes (1)
Axe	No (0)	Yes (1)
Hoe	No (0)	Yes (1)
Shovel	No (0)	Yes (1)
Sickle	No (0)	Yes (1)
Modern beehive	No (0)	Yes (1)
Traditional beehive	No (0)	Yes (1)
**Housing characteristics**		
Indoor plumping/ pipe water	No (0)	Yes (1)
Type of flooring	Earth/dung (0)	Cement/raw wood (1)
Toilet facility	Unsanitary or traditional pit latrine/ no toilet (0)	Sanitary or improved pit latrine (1)
**Other household materials**		
Sofa	No (0)	Yes (1)
Bed	No (0)	Yes (1)
Table	No (0)	Yes (1)
Chair	No (0)	Yes (1)

Stove	No (0)	Yes (1)

Part IX: Household (Point of use) Water Sample Collection

Household ID ________________________	Date collected _______________
Name of Kebele __________________	Time collected ______________
Village Name _________________________	
Water source name ___________________	
Water sample code ___________________	

Name of data collector _________________________ Signature _______________

Part X: Water Sample Collection from Water Sources

Water Source Name ___________________	Date collected _______________
Name of Kebele __________________	Time collected ______________
Village Name _________________________	
Water sample code ___________________	

Name of Sample collector _________________________ Signature _______________

Part XI: Laboratory result reporting format

No.	Type of Test	Result	Remark
**1.**	**Membrane Filtration**	**_______ Thermotolerant Coliform colonies/100ml**	

Name of laboratory investigator________________________

Signature: _____________

Date: _____________
