# Supplementary material for: Bacteriological quality of drinking water from source and point of use and associated factors among households in Eastern Ethiopia
Source: PLoS One. 2021 Oct 15;16(10):e0258806. doi: 10.1371/journal.pone.0258806 (PMC8519474; doi:10.1371/journal.pone.0258806)
Supplement: S2 File — (PDF) [file pone.0258806.s005.pdf]

የአማርኛመጠይቅ

ይህ መጠየቅ የመጠጥ ውሃ ንጽህና ከጀርም (ተዋህስያን) አንጻር ከመነሻ እስከ መኖሪያ ቤት ያለውን ልዩነት እና አጋላጭ ምክንያቶችን ለማጥናት የተዘጋጀ መጠይቅ ነው።

ቀበሌ ____________________ ኮድ_________________

መረጃ ሰብሳቢው ስም ______________________ ፊርማ _______ ቀን __________________

ክፍል አንድ፡- የማህበራዊ እና ኢኮኖሚያዊ ሁኔታ

የጥያቄ ቁጥር	ጥያቄ	የጥያቄምርጫ	ዝለል
101	የቤተሰብ ሀላፊ	1. ወንድ<br>2. ሴት	
102	ዕድሜዎ ስንት ነው?	______________	
103	የተሳታፊ ፆታ	1. ወንድ<br>2. ሴት	
104	ብሄር	1. ኦሮሞ<br>2. አማራ<br>3. ሃረሪ<br>4. ጉራጌ<br>5. ሌሎችካሉይጠቀስ ___________	
105	ሐይማኖት	1. ሙስሊም<br>2. ኦርቶዶክስ ክርስቲያን<br>3. ፕሮቴስታንት (ፔንጤ)<br>4. ሌላ ካለ ይጥቀሱ____________	
106	የት/ት ደረጃዎ ስንት ነው?	1. ማንበብና መጻፍ የማይችሉ<br>2. ማንበብና መጻፍ የሚችሉ (መደበኛ ያልሆነ ትምህርት)<br>3. አንደኛ ደረጃ (1-8ኛክፍል) ያጠናቀቀ	

		4. ሁለተኛ ደረጃ (9-12 ክፍል) ያጠናቀቀ<br>5. ኮሌጅ እና ከዛበላይ	
107	የጋብቻሁኔታ	1. ያላገቡ<br>2. ያገቡ<br>3. ፍቺ የፈፀሙ<br>4. ባሏየሞተባት<br>5. ሌላካለይጥቀሱ_______________	
108	መደበኛ ስራዎ ምንድነው?	1. ንግድ<br>2. መንግስትሰራተኛ<br>3. የቀን/ የጉልበትሠራተኛ<br>4. ሥራ-አጥ / እየሰራ አይደለም (ጡረተኛ)<br>5. ሌላካለይጥቀሱ ______________	
109	የቤተሰብ ብዛት	________________	
110	የቤቱ መገኛ	1. ገጠር 2. ከተማ	

ክፍል ሁለት፡ የመጠጥ ውሃ አቅርቦት ሁኔታ በሚመለከት

201	የቤተሰቡ የመጠጥ ዉሀ መገገኛ ምንጭ ከየት ነዉ?	1. የቧንቧ ውሃ በመኖሪያ ቤት ውስጥ<br>2. የቧንቧ ውሃ በግቢ ውስጥ<br>3. የህዝብ ቧንቧ /ቦኖ/<br>4. የተጠበቀ የጉድጓድ ውሃ<br>5. የተጠበቀ የምንጭ ውሃ<br>6. ሌላ ካለ ይጥቀሱ __________	1 ወይም 2 ከሆነ ወደ ጥያቄ 204
202	ከውሃው መገኛ ውሃ ለማምጣት ምን ያህል ጊዜ ይፈጃል?	__________ (በደቂቃ)	

203	ውሃውን ከመገገኛ ቦታው ቀድቶ የሚያመጣው ማነው?	1. አዋቂ ሴት<br>2. አዋቂ ወንድ<br>3. ሴቶች ህፃናት (ከ15አመትበታች)<br>4. ወንዶች ህፃናት (ከ15አመትበታች)	
204	በቀን ውስጥ በአማካይ አንድ ሰው ምን ያህል ውሃ ይጠቀማል?	___________________ (በሊትር)	
205	ለውሃ አገልግሎት ይከፍላሉ?	1. አዎ<br>2. አይ	

ክፍል ሦስት፡ የመጠጥ ውሃ አቀማመጥና አያያዝ ሁኔታ

301	በምን ያህል ጊዜ የመጠጥ ውሃ ትቀዳላችሁ?	1. በየቀኑ<br>2. በየሁለት ቀን ልዩነት<br>3. በየሦስት ቀን ልዩነት<br>4. በየሳምንቱ<br>5. ሌላ ካለ ይጥቀሱ __________	
302	ለምን ያህል ጊዜ የመጠጥ ውሃን ታስቀምጣላችሁ?	_________(ቀናት)	
303	የመጠጥ ውሃን በምን ውስጥ ታስቀምጣላችሁ?	1. ጀሪካን<br>2. ባልዲ<br>3. ሌላካለይጥቀሱ __________	
304	በጉብኘቱ ወቅት የመጠጥ ውሃ ማጠራቀሚያው ክዳን አለው? (በምልከታ)	3. አዎ<br>4. አይ	
305	የመጠጥ ውሃ ማጠራቀሚያው በየጊዜው ይታጠባል?	1. አዎ<br>2. አይ	2 ከሆነወደጥያቄ 307

306	በየምን ያህል ጊዜ የውሃ ማጠራቀሚያችሁን ታጥባላችሁ?	1. በየቀኑ<br>2. በየሦስትቀኑ<br>3. በየሳምንቱ<br>4. በየአስራአምስትቀኑ<br>5. በጭራሽ<br>6. ሌላካለይጥቀሱ __________	
307	የመጠጥ ውሃን እንዴት ከማጠራቀሚው ትቀዳላችሁ?	1. በማንቆርቆር<br>2. በመጥለቅ	1 ከሆነወደጥያቄ 309
308	በመጥለቅ ከሆነ መጥለቂያው መያዣ አለው?	1. አዎ<br>2. አይ	
309	የመጠጥ ውሃ ማጠራቀሚያውን ልጆች ይደርሱበታል?	1. አዎ<br>2. አይ	
310	የውሃ መጠጫችሁን የት ታስቀምጣሊችሁ?	1. በወለል ላይ<br>2. ለውሃ መጠጫ ማስቀመጫ በተዘጋጀ ቦታ<br>3. በውሃ ማጠራቀሚያው ላይ ሌላካለይጥቀሱ ______________	

ክፍል አራት፡ የፅዳት ሁኔታ

401	ቤተሰቡ የትገኛውን አይነት የመፀዳጃ አገልግሎት ይጠቀማል?	1. በውሃ የሚሰራ መፀዳጃ ቤት<br>2. አየር ማስወጫ ያለው መፀዳጃ ቤት<br>3. አየር ማስወጫ የሌለው መፀዳጃ ቤት<br>4. ምንም የለም (ሜዳ ላይ)<br>5. ሌላ ካለ ይጥቀሱ __________	

402	መፀዳጃ ቤቱን ሌሎች ቤተሰቦች በጋራ ይጠቀማሉ?	1. አዎ<br>2. አይ	2 ከሆነወደጥያቄ 404
403	ምን ያህል ቤተሰቦች መፀዳጃ ቤቱን ይጋራሉ?	__________ ቤተሰብ	
404	የህፃናትን አይነ-ምድር እንዴት ያስወግዱለ?	1. ህፃናቱ መፀዲጃ ቤት ይጠቀማል<br>2. መጸዲጃ ቤት ውስጥ በመዴፊት<br>3. ሜዲሊይ<br>4. ከላሊ ቆሻሻ ጋር በመድፋት<br>5. ሌላ ካለ ይጥቀሱ ____________	
405	ደረቅ ቆሻሻን እንዴት ታስወግዲላቸሁ?	1. ሜዲ ሊይ<br>2. በመቅበር<br>3. በማቃጠል<br>4. እንዲበሰብስ በማዴረግ	
406	ከቤት የሚወጣውን ፍሳሽ እንዳት ታስወግዲላችሁ?	1. ጉድጓድ ውስጥ በመጨመር<br>2. በመፀዲጃቤትውስጥበመጨመር<br>3. ሜዲ ላይ በማፍሰስ<br>4. ላሊ ከሆነ ይጥቀሱ ___________	
407	በመኖሪያ ቤቱ ውስጥ እንስሳት አብረው ይኖራሉ?	1. አዎ<br>2. አይ	

ክፍል አምስት፡ የንፅህና ሁኔታ

501	ውሃ ከመቅዳትዎ በፊት እጅዎን ይታጠባሉ?	3. አዎ<br>4. አይ	
502	ከመፀዳጃ ቤት መልስ እጅዎን ይታጠባሉ	1. አዎ<br>2. አይ	2 ከሆነወደጥያቄ 504
503	የሚታጠቡ ከሆነ ሳሙና ይጠቀማሉ	1. አዎ<br>2. አይ	
504	ህጻናትን ካጠቡ በሁዋላ እጅዎን ይታጠባሉ?	1. አዎ<br>2. አይ	2 ከሆነወደጥያቄ 506
505	የሚታጠቡ ከሆነ ሳሙናይ ጠቀማሉ?	1. አዎ<br>2. አይ	
506	ህፃናትን ከመመገቦ በፉት እጅዎን ይታጠባለ?	1. አዎ<br>2. አንዲንዴጊዜ<br>3. አይ	3 ከሆነወደጥያቄ 508
507	የሚታጠቡ ከሆነ ሳሙና ይጠቀማሉ?	1. አዎ<br>2. አይ	
508	በግቢው ውስጥ የእጅ መታጠቢያ ቦታ አለ? (በምልከታ)	1. አዎ<br>2. አይ	2 ከሆነወደጥያቄ 601
509	ካለ የእጅ መታጠቢያው ውሃ አለው? (በምልከታ)	1. አዎ<br>2. አይ	
510	ሳሙና ወይም ሌሎች ፅዳትን መጠበቂያዎች እንደ አመድ፣ አሸዋ አሉ? (በምልከታ)	1. አዎ<br>2. አይ	

ክፍል ስድስት፡ የቤት ውስጥ የመጠጥ ውሃ ህክምና

601	በቤት ውስጥ የመጠጥ ውሃ ህክምና ይጠቀማሉ	1. አዎ<br>2. አይ	2 ከሆነ ወደጥያቄ 701
602	የሚተቀሙ ከሆነ የትኛውን መንገድ ይጠቀማሉ	1. ክሎሪን በመጨመር<br>2. በንፁህ ጨርቅ በማጥለል<br>3. ለተወሰነ ጊዜ አስቀምጦ በማዝቀጥ<br>4. በማፍላት<br>5. ሌላ ካለ ይጥቀሱ __________	

ክፍል ሰባት፡ ለውሃ መበከል አስተዋፅኦ የሚያደርጉ ምክንያቶች ላይ ያለ ግንዛቤ

70<br>1	የውሃ ብክለት የት ሊከሰት ይችላል ብለው ያስባሉ	1. ከመነሻቦታ<br>2. በቤትውስጥ<br>3. በሁለቱም<br>4. ሌላካለይጥቀሱ __________	
70<br>2	የውሃ መቅጃና ማጠራቀሚያን ማጠብ የውሃ ብክለትን ይከላከላል ብለው ያስባሉ	1. አዎ<br>2. አይ	
70<br>3	የመጠጥ ውሃን ከመቅዳት በፊት እጅን በውሃና በሳሙና መታጠብ የውሃ ብክለትን ይከላከላል ብለውያስባሉ	1. አዎ<br>2. አይ	
70<br>4	ከመፀዳጃ ቤት መልስ እጅን በውሃና በሳሙና መታጠብ የውሃ ብክለትን ይከላከላል ብለውያስባሉ	1. አዎ<br>2. አይ	
70<br>5	መፀዳጃ ቤትን በአግባቡ መጠቀም የውሃ ብክለትን ይከላከላል ብለው ያስባሉ	1. አዎ<br>2. አይ	
70<br>6	ውሃን በቤት ውስጥ ማከም የውሃ ብክለትን ይከላከላል ብለው ያስባሉ	1. አዎ<br>2. አይ	

707	ባለፉት 3 ወራት ውስጥ ስለ ውሃ አቅርቦት እና ንጽህና በተመለከተ ትምህርት አግኝተው ያውቃለ?	1. አዎ<br>2. አይ	

ክፍል ስምንት፡ የቤተሰብ የሀብት ጠቋሚ ምክንያቶች

ከሚከተሉት ውስጥ የትኛው በቤትዎ ውስጥ ይገኛል?	ምላሽ	
በሬ	የለም (0)	አለ (1)
ላም	የለም (0)	አለ (1)
ጥጃ	የለም (0)	አለ (1)
በግ	የለም (0)	አለ (1)
ፍየል	የለም (0)	አለ (1)
ፈረስ	የለም (0)	አለ (1)
አህያ	የለም (0)	አለ (1)
ዶሮ	የለም (0)	አለ (1)
**ቋሚንብረት**		
ቴሌቭዥን	የለም (0)	አለ (1)
ራዲዮ	የለም (0)	አለ (1)
መብራት	የለም (0)	አለ (1)
ፍርጅ	የለም (0)	አለ (1)
የቤትስልክ	የለም (0)	አለ (1)
ተንቀሳቃሽስልክ	የለም (0)	አለ (1)
መኪና	የለም (0)	አለ (1)
ሞተርሳይክል	የለም (0)	አለ (1)
ሳይክል	የለም (0)	አለ (1)
ጋሪ	የለም (0)	አለ (1)
ወርቅ	የለም (0)	አለ (1)

የራስሽ(ዎ) መኖሪያቤት	የለም (0)	አለ (1)
የራስሽ(ዎ) የእርሻመሬት	የለም (0)	አለ (1)
**የማምረቻዕቃዎች**		
ማረሻ	የለም (0)	አለ (1)
መጥረቢያ	የለም (0)	አለ (1)
መኮትኮቻ	የለም (0)	አለ (1)
አካፋ	የለም (0)	አለ (1)
ማጭድ	የለም (0)	አለ (1)
ዘመናዊየንብቀፎ	የለም (0)	አለ (1)
ባህላዊየንብቀፎ	የለም (0)	አለ (1)
**የቤቱሁኔታ**		
በቤትውስጥየውሀቧንቧ	የለም (0)	አለ (1)
የወለልአይነት	አፈር (0)	ሲሚንቶ/ እንጨት (1)
የመፀዳጃ ቤት አይነት	ንፅህናውን ያልጠበቀ/ አየር ማስወጫ የሌለው(0)	ንፅህናውን የጠበቀ/ አየር ማስወጫ ያለው (0)
**የቤትቁሳቁስ**		
ሶፋ	የለም (0)	አለ (1)
አልጋ	የለም (0)	አለ (1)
ጠረንጴዛ	የለም (0)	አለ (1)
ወንበር	የለም (0)	አለ (1)
እስቶቭ	የለም (0)	አለ (1)

ክፍል ዘጠኝ፡ ከተመረጡ ቤቶች የውሃ ናሙና መሰብሰቢያ ፎርማት

የመኖሪያ ቤቱ ኮድ ___________________	የተሰበሰበበት ቀን _________________
ቀበሌ _____________________	የተሰበሰበበት ሰዓት________________
መንደር _______________________	
የውሃው መገኛ ስም____________________	
የውሃው ናሙና ኮድ ___________________	

የሰብሳቢው ስም ___________________________________ ፊርማ ______________________

ክፍል አስር፡ የውሃ መነሻዎች የውሃ ናሙና መሰብሰቢያ ፎርማት

የውሃው መገኛ ስም___________________	የተሰበሰበበት ቀን _________________
ቀበሌ _____________________	የተሰበሰበበት ሰዓት________________
መንደር _______________________	
የውሃው ናሙና ኮድ ___________________	

የሰብሳቢው ስም ___________________________________ ፊርማ ______________________

ክፍል አስራ አንድ - የላቦራቶሪ ውጤት ሪፓርት ፎርማት

No.	የሙከራ አይነት	ውጤት	አስተያየት
1.	Membrane Filtration (ውሃን በማጣራት)	________ Thermotolerant Coliform colonies/100ml	

የላብራቶሪ መርማሪ ስም ______________________

ፊርማ ______________________

ቀን ______________________
