# Supplementary material for: Bacteriological quality of drinking water from source and point of use and associated factors among households in Eastern Ethiopia
Source: PLoS One. 2021 Oct 15;16(10):e0258806. doi: 10.1371/journal.pone.0258806 (PMC8519474; doi:10.1371/journal.pone.0258806)
Supplement: S3 File — (PDF) [file pone.0258806.s006.pdf]

Gaaffilee Afaan Oromoon qophaa'an

Gaaffileen kun waa'ee qorannoo qabiyyee qulqullinqa bishaaniif jarmiilee dhukkuba namatti fidanii fi isa waliin walqabatanii wantoota jiranii fi haala bishaanitti fayyadaman bakka madda isaa hanga itti fayyadamanitti qorachoochuuf kan qophaa'e dha.

Kebele _______________ lakk. adda baasii abbaa warraa (ID)_______________

maqaa nama odeeffanoo funaanuu ___________________ mallattoo________ guyyaa________

kutaa 1ffaa: waa'ee eenyummaa nama gaaffatamee kan mullisu

lakk	Gaaffii	Deebii	Irra darbi
101	Abbaa warraa mana	1. Dhiira<br>2. Dhalaa	
102	Umurii kee meeqa ?	Waggaa---------------	
103	Abbaa warraa deebii kenne	1. Dhiira<br>2. Dhalaa	
104	Qomoo kee maali?	1. Oromoo<br>2. Amaara<br>3. Hararii<br>4. Guraagee<br>5. Kan Biraa _________________	
105	Amantiin kee maali?	1. Musliima<br>2. Ortoodoksii<br>3. Protestaantii<br>4. Kan Biraa ________________	
106	Sadarkaa barnootaa	1. Dubbisuuf Barressuu Kan Hin Dandeenye<br>2. Dubbisuuf Barressuu Kan Dandahuu<br>3. Kutaa 1-8	

		4. Kutaa 9-12<br>5. Kolleejjii Fi Isaa Ol	
107	Haala gaa'elaa	1. Hin Fuudhiin/Hin Eerumiin<br>2. Kan Fuude Ykn Heerumte<br>3. Kan Hiike /Hiikte<br>4. Kan Irraa Du'e<br>5. Kan Biraa ____________	
108	Hojiin kee maali?	1. Daldalaa<br>2. Hojjetaa Mootummaa<br>3. Kan Hin Qacaramne/Kan Hojii Hin Qabne/Soorama Kan Bahe<br>4. Dafqaan Bulaa<br>5. Kan Biraa __________	
109	Lakkoofsa maatii mana kana keessa jiraatanii?	Lakk _______________	
110	Mana jirenyaa	1. Badiyaa 2. Magaalaa	

Kutaa 2ffaa: Gosa madda bishaanii

201	Bishaan mana kana keessatti fayyadamtan eessaa argattu?	1. Bishaan Boombaa Mana Biraa<br>2. Bishaan Boombaa Ooyruu<br>3. Boombaa Magaalaa (Bonoo)<br>4. Bishaan Boollaa Eegamu Irraa<br>5. Laga Burqaa Eegamu Irraa<br>6. Kan Biraa __________	Yoo 1 ykn 2 tahe, gara gaaffii 204

202	Bishaan deemtanii fiduuf dhaquuf galuuf daqiiqaa meeqa fudhata?	Daqiiqaaa ___________	
203	Bishaan kana eenyutu fida?	1. Dardara Dhiira<br>2. Dardara Dhalaa<br>3. Ijoollee Dhiiraa (Wagaa 15 Gadii)<br>4. Ijoollee Durbaa (Wagaa 15 Gadii)	
204	Guyyaan tokko kessaa nama tokko biishaan hangam fayyadamaa?	____________ litra	
205	Bishaanif nikafaltanii	1. Eyyee 2. Lakkii	

Kutaa 3ffaa: Mana keessatti akkaataa bishaan itti taa uu fi itti qabamu

301	Bishaan si'a meeqa fidda ?	1. Guyyaa Guyyaatti<br>2. Guyyaa Tokko Oolee<br>3. Guyyaa Sadii Booda<br>4. Torbeetti<br>5. Kan Biraa ___________	
302	Bishaan dhugaatii guyyaa meeqaaf mana keessa?	Guyyaa ___________	
303	Man keessatti bishaan dhugaatii maal keessa keessa	1. Jarkaana (Mikaa)<br>2. Baaldii<br>3. Kan Biraa __________	
304	Meeshaan itti bishaan keessu qadaada qabaa?(ilaali)	2. Eyyee<br>3. Lakkii	
305	Bishaan meeshaa itti waraabduuf itti keewwattu ni dhiqxaa?	1. Eyyee<br>2. Lakkii	Yoo 2 tahe, gara gaaffii

			307
306	Qodaa bishaan keessa keessu si a meeqa dhiqxa?	1. Guyyaa Guyyaatti<br>2. Guyyaa Sadii Booda<br>3. Torbeetti Al Takka<br>4. Guyyaa 15 Al Takka<br>5. Hin Dhiqu Lakki<br>6. Kan Biraa __________	
307	Qodaa bishaan keessa keessu keessaa akkamitti qodaa biraatti waraabda ?	1. Ittiin Naqa<br>2. Keesa Kaa'een Waraaba	Yoo 1 tahe, gara gaaffii 309
308	Bishaan keesa kaa'een waraaba yo tae meshaa kan itiin waraabtan haarka niqabaa?	1. Eyyee<br>2. Lakkii	
309	Bakka bishaan kaa amu daa imni gahuu dandeessii?	3. Eyyee<br>4. Lakkii	
310	Meeshaa itti dhugdan (qalasa) eessa keessan?	1. Laffa irraa<br>2. Bakka itti qophaayeef<br>3. Bakka bishaan itti keessan	

Kutaa 4ffaa: Waa'ee qulqullinaa

401	Mana fincaanii gosa kam fayyadamtu?	1. Bishaan Itti Naqamee Kan Dhiqamu<br>2. Mana Fincaanii Hurka Baasu<br>3. Mana Fincaanii Boolla Qadaada Qabu<br>4. Mana Fincaanii Hin Qabnu (Oyruu)<br>5. Kan Biraa __________	

402	Mana fincaanii keessan olla waliin fayyadamtuu?	1. Eyyee<br>2. Lakkii	Yoo 2 tahe, gara gaaffii 404
403	Deebiin gaffii 402 Eyyee yoo tahe Mana meeqatu mana fincaanii keessan sin walliin fayyadama?	___________________	
404	Boilli daa'imani eesatii darbitan?	1. Daaiman mana fincaanii nifayadamaa<br>2. Mana fincaanii keessatii naqu<br>3. Lafa irratii<br>4. Koosii wajiin darbudhaan<br>5. Kan Biraa _________________	
405	Koosii (balfa gogogaa) eessattii nayxan?	1. Lafa irrattii<br>2. Bola koosii keessatti<br>3. gubudhaan<br>4. koossii akka tortoru godhdhan itti fayyadamu<br>5. Kan Biraa _________________	
406	Balfa dhangal'oo eessattii naqtan?	1. Boola keessaattii<br>2. Mana fincaanii keessaattii<br>3. Lafa irrattii ganaqudhaan	
407	Mana ittii jirtan keessa beeyladooni ni jiraa? (wajiin galaa)	1. Eyyee 2. Lakkii	

Kutaa 5ffaa: Qulqullina eeggachuu

501	Bishaan waraabuun dura harka ni dhiqattaa?	1. Eyyee<br>2. Lakkii	Yoo 2 tahe, gara gaaffii 503
502	Mana fincaanii erga fayyadamtee booda harka ni dhiqattaa?	1. Eyyee 2. Lakkii	Yoo 2 tahe, gara gaaffii 504

503	Deebiin kee eyyee yoo ta’e yogguu harka dhiqattu saamunaa ni fayyadamtaa?	1. Eyyee 2. Lakkii	
504	Ijollee erga dhiqxan booda harka ni dhiqattaa?	1. Eyyee 2. Lakkii	Yoo 2 tahe, gara gaaffii 506
505	Deebiin kee eyyee yoo ta’e yogguu harka dhiqattu saamunaa ni fayyadamtaa?	1. Eyyee 2. Lakkii	
506	Ijoollee osoo hinnyaachisin dura harka ni dhiqataa?	1. Eyyee<br>2. Hogu takka takka<br>3. Lakkii	Yoo 3 tahe, gara gaaffii 508
507	Deebiin kee eyyee yoo ta’e yogguu harka dhiqattu saamunaa ni fayyadamtaa?	1. Eyyee 2. Lakkii	
508	Bakka harka itti dhiqatan niqabdanii? (ilaali)	1. Eyyee 2. Lakkii	Yoo 2 tahe, gara gaaffii 601
509	Deebiin kee eyyee yoo ta’e bishaan niqabaa? (ilaali)	1. Eyyee 2. Lakkii	
510	Saamunaa yoo kin darraa ni jiraa? (ilaali)	1. Eyyee 2. Lakkii	

Kutaa 6ffaa: Mala bishaan ittiin qulqulleessinu

601	Maatiin kun malloota bishaan ittiin qulqulleessan ni fayyadamaa?	1. Eyyee<br>2. Lakkii	
602	Yoo deebiin kee eyyee tahe malloota bishaan itti qulqulleessan isa kam fayyadamtu?	1. Kiloorinii Itti Naquu<br>2. Calaluu<br>3. Kaa’anii Tursuu<br>4. Danfisuu<br>5. Kan Biraa _______________	

Kutaa 7ffaa: Beekumsa faalama bishaanii

701	Bishaan dhugaatii eessatti faalama jettee yaadda?	1. Bakka Waraabamutti<br>2. Mana Keessatti<br>3. Bakka Lachuu<br>4. Kan Biraa _____________	
702	Meeshaa bishaan keessa kaa’amuu fi itti waraabamu qulqulleessuun faalama bishaanii ittisa jettee yaaddaa?	1. Eyyee 2. Lakkii	
703	Bishaan waraabuun dura harka dhiqachuu faalama bishaanii ittisa jettee yaaddaa?	1. Eyyee 2. Lakkii	
704	Mana fincaanii fayyadamuun booda harka dhiqachuun faalama bishaanii ni ittisa jettee yaaddaa?	1. Eyyee 2. Lakkii	
705	Mana fincaaniitti sirnaan fayyadamuun faalama bishaanii ni hambisa jettaa yaaddaa?	1. Eyyee 2. Lakkii	
706	Mana keessatti bishaan qulqulleessuun faalama bishaanii ni hambisa jettee yaaddaa?	1. Eyyee 2. Lakkii	
707	Baatii sadeen dabree keessaa waa’ee bishaani fi qulqulina bartanii jirtu?	1. Eyyee 2. Lakkii	

Kutaa 8ffaa: Gaaffiilee Haala Qabeenya Maatii Ilaallatan

Kanneen armaan gadii keessaa warreen kamtu mana keessaniitti argamaa?

Gosa qabeenyaa	Deebii	
**Beeylada**		
Sangaa	Lakki (0)	Eeyyeen (1)
Sa'aa	Lakki (0)	Eeyyeen (1)
Jabbii	Lakki (0)	Eeyyeen (1)
Hoolaa	Lakki (0)	Eeyyeen (1)
Re'ee	Lakki (0)	Eeyyeen (1)
Farda	Lakki (0)	Eeyyeen (1)
Harree	Lakki (0)	Eeyyeen (1)
Lukkuu	Lakki (0)	Eeyyeen (1)
**Meeshaalee dhaabbataa**		
Televizinii	Lakki (0)	Eeyyeen (1)
Raadiyoonii	Lakki (0)	Eeyyeen (1)
Elektirikii	Lakki (0)	Eeyyeen (1)
Frijii	Lakki (0)	Eeyyeen (1)
Bilbila manaa	Lakki (0)	Eeyyeen (1)
Mobaayilii	Lakki (0)	Eeyyeen (1)
Konkolaataa	Lakki (0)	Eeyyeen (1)
Motarsaayikilii	Lakki (0)	Eeyyeen (1)
Saaykilii	Lakki (0)	Eeyyeen (1)
Gaarii	Lakki (0)	Eeyyeen (1)
Faaya (warqee, qarshii)	Lakki (0)	Eeyyeen (1)
Mana jireenyaa dhuunfaa	Lakkii (0)	Eeyyeen (1)
Lafa qotisaa	Lakki (0)	Eeyyeen (1)
**Meeshaalee omishaa**		
Maarashaa	Lakki (0)	Eeyyeen (1)
Qottoo	Lakki (0)	Eeyyeen (1)
Doomaa	Lakki (0)	Eeyyeen (1)

Akaafaa	Lakki (0)	Eeyyeen (1)
Hamtuu	Lakki (0)	Eeyyeen (1)
Gaagura kaannisaa kan amayyaa	Lakki (0)	Eeyyeen (1)
Gaagura kaannisaa kan aadaa	Lakki (0)	Eeyyeen (1)
**Haala mana jireenyaa**		
Bishaan ujummoodhan argamu	Lakki (0)	Eeyyeen (1)
Lafa mana keessaa	Biyyee (0)	Cimintoo/ xaawullaa (1)
Haala mana fincaanii	Qulqullina kan hin qabne/ gutumaatti kan hin qabne (0)	Kan qulqullina qabu (1)
**Meeshalee manaa biroo**		
Soofaa	Lakki (0)	Eeyyeen (1)
Siree	Lakki (0)	Eeyyeen (1)
Xarabeezzaa	Lakki (0)	Eeyyeen (1)
Taa'umsa	Lakki (0)	Eeyyeen (1)
Istoovii	Lakki (0)	Eeyyeen (1)

Kutaa 9ffaa: Formatii bishaan mana iraa kan fudhamee

lakk. adda baasii abbaa warraa (ID) ________________	Guyyaa_______________
Aradaa__________________	Sa'a ______________
Ganda____________________	
Maqaa Bishaan Madda_________________	
Coodii Bishaan________________	

Maqaa ____________________________ Mallattoo _______________

Kutaa 10ffaa: Formatii bishaan Madda iraa kan fudhuu

Maqaa Bishaan Madda ________________	Guyyaa _______________
Aradaa __________________	Sa'a ______________
Ganda _________________________	
Coodii Bishaan ___________________	

Maqaa ___________________________ Mallattoo _______________

Kutaa 10ffaa: Debii Laboraatoorrii Irra Argamee

Lak.	Goosa Yaalii	Debii	Yaada
**1.**	**Membrane Filtration**<br>**(Biishaan Calaluu)**	**_______ Thermotolerant Coliform colonies/100ml**	

Maqaa ___________________

Mallattoo________

Guyyaa________
